# A Rare Case of Perforated Descending Colon Cancer Complicated with a Fistula and Abscess of Left Iliopsoas and Ipsilateral Obturator Muscle

**DOI:** 10.1155/2014/128506

**Published:** 2014-03-16

**Authors:** Alban Cacurri, Gaspare Cannata, Stefano Trastulli, Jacopo Desiderio, Antongiulio Mangia, Olga Adamenko, Eleonora Pressi, Giorgio Giovannelli, Giuseppe Noya, Amilcare Parisi

**Affiliations:** ^1^Department of General and Oncologic Surgery, University of Perugia, 06157 Perugia, Italy; ^2^Department of Digestive and Liver Surgery Unit, St. Maria Hospital, 05100 Terni, Italy

## Abstract

Perforation of descending colon cancer combined with iliopsoas abscess and fistula formation is a rare condition and has been reported few times. A 67-year-old man came to our first aid for an acute pain in the left iliac fossa, in the flank, and in the ipsilateral thigh. Ultrasonography and computed tomography revealed a left abdominal wall, retroperitoneal, and iliopsoas abscess that also involved the ipsilateral obturator muscle. It proceeded with an exploratory laparotomy that showed a tumor of the descending colon adhered and perforated in the retroperitoneum with abscess of the iliopsoas muscle on the left-hand side, with presence of a fistula and liver metastases. A left hemicolectomy with drainage of the broad abscess was performed. Pathologic report findings determined adenocarcinoma of the resected colon.

## 1. Introduction

Infections of the retroperitoneal space have troubled physicians in all disciplines for decades because of remarkable diagnosis and treatment problems along with a mortality rate which is around 20% [1]. Retroperitoneal abscesses with no triggering cause are rare. In the past the most frequent were the “cold” ones as tuberculosis, whereas today the “hot” ones, secondary to Crohn's disease, pyelonephritis, and diverticulitis, are most frequent [2]. It is very rare that a retroperitoneal abscess, particularly in the iliopsoas muscle, is caused by a perforation of a cancer of the descending colon.

In fact colon cancer, which represents the most frequent tumor of the digestive tract [3], involves bleeding, perforative, and occlusive events among the most common complications; these latter ones are more suggestive of a tumor of the descending colon. Furthermore the invasion of adjacent organs can be found frequently in case of cancer of the left colon in an advanced stage like a T4 according to the TNM classification and staging of the 2010 [4]. But an event like the perforation with invasion of the abdominal wall and the possible formation of an abscess in the retroperitoneal space is relatively rare [5]. In cases where a colorectal cancer shows atypical clinical findings the diagnosis is very difficult; moreover, the delay of the diagnosis and the inadequate clinical management represent a major cause of an increase in morbidity and mortality [5–8].

Here is a case of cancer of the descending colon which was presented with an unusual abscess of the left iliopsoas muscle and ipsilateral obturator muscle.

## 2. Case Report

A 67-year-old man came to our first aid for an acute pain in the left iliac fossa, in the flank, and in the ipsilateral thigh. He had a 5-day history of pain in walking and in the left lower limb.

At ER the patient showed no pathological vital parameters, with the exception of a body temperature of 37.5°C.

The physical examination showed an abdominal distension localized in left iliac fossa and flank. A deep painful mass of stretched-elastic consistency was palpated on the left lower quadrant, anything of relevance in the remaining abdominal quadrants. The sign of Blumberg was negative. The femoral wrists were present.

The clinical suspicion was a diverticulitis of sigma complicated by a perforation and peridiverticular abscess (Hinchey IIa-IIb). Then were performed laboratory hematobiochemical routine tests and we proceeded with diagnostic imaging: an abdominal ultrasound and CT with and without iodate contrast.

On admission in ER the following hematobiochemical values were altered: the WBC of 21.23∗10^3^/mm^3^ (n.v. 4.50–10.80), with remarkable neutrophilia: 87.1% (n.v. 40.5–74.1%); C-reactive protein of 22 mg/dL (n.v. < 0.1 mg/dL); the erythrocyte sedimentation rate of 35 mm/h (n.v. 0–20 mm/h).

**Figure 1:**
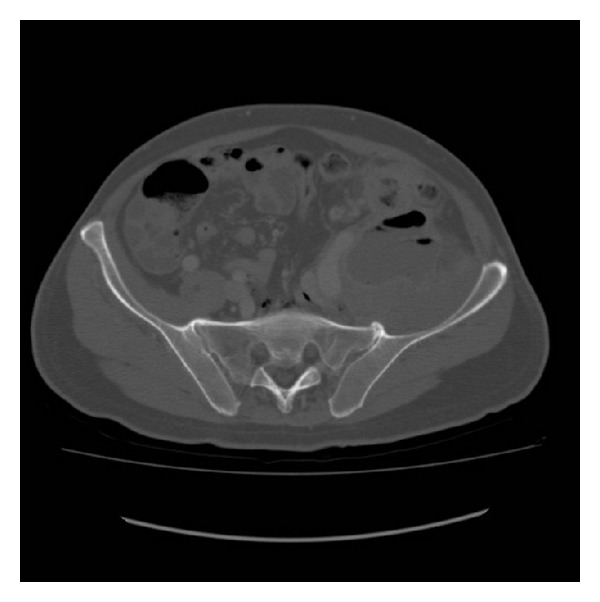


**Figure 2:**
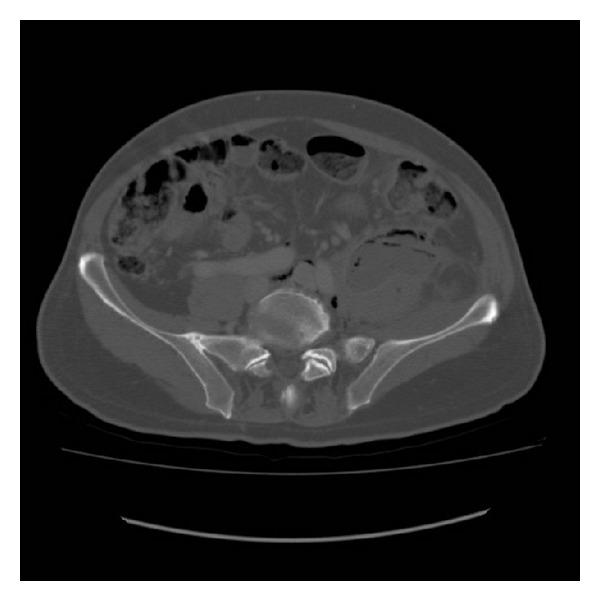


The abdominal ultrasonography showed a thickening of the colic walls in left flank-iliac fossa with hyperdense appearance of perivisceral fat and neighboring fluid. We proceed to a computerized axial tomography performed with and without iodate contrast. The CT (Figures 1 and 2) showed a liver increased in volume, with focal lesions of probable abscessual nature and a big abscess in the left psoas muscle, that appeared to be increased in volume and that also involved the ipsilateral obturator muscle.

So was performed an emergency intervention. It proceeded with an exploratory laparotomy which emphasized left dolichocolon. It showed a tumor of the descending colon adhered and perforated in the retroperitoneum with abscess of the iliopsoas muscle on the left-hand side, with presence of a fistula and liver metastases. So was performed a left hemicolectomy with tying of the mesenteric artery below the origin, removal of the tablet seat of peritoneal perforation, drainage of the broad abscess of the iliopsoas muscle, removal of the pus to culture examination, and the washing of the abscessual cavity with double drainage of the same. A metastasis surfacing the IV hepatic segment was asportated.

The culture examination of the abscessual liquid was positive for the following bacteria:* Citrobacter braakii* and* Escherichia coli*. After antibiogram, the patient was treated with adequate targeted antibiotic therapy. The results of histopathological examination of the removed colic mass confirmed the diagnosis of adenocarcinoma with the following TNM classifications [4]: T4 N2 M1 and G3, according to the grading drawn up by the WHO. The patient had a regular postoperative progress with resolution of symptoms and is currently undergoing adjuvant chemotherapy.

## 3. Discussion and Conclusions

The first abscess of the psoas was described by Mynter in 1881; he was talking about this clinical condition using the term “psoitis” [9]. In general, the abscesses of the psoas muscles can be classified into primary or secondary depending on the presence or not of an underlying pathology. The primary abscesses occur as a result of a spread by the hematogenic via of an infectious process (Table 1), while Crohn's disease represents the most common cause of secondary abscess of the psoas [10]. Other common secondary causes of abscess of the psoas muscles (Table 2) comprise more than Crohn's disease (60% of cases), the appendicitis (16%), ulcerative colitis, and diverticulitis (11%) [11]. In a review of 367 cases, Ricci et al. noticed a different etiology of the abscess of the psoas muscles depending on the geographical location [12]; in Asia and Africa more than 99% of the psoas abscesses are primary, while in Europe and in North America primary abscesses are, respectively, 17% and 61% [12]. The iliopsoas abscess is more common in the young than in the elderly [13]. It was also reported to be more common in boys than in girls [14, 15]. The mortality rate in primary iliopsoas abscesses is around 2.4%, while 19% in secondary abscesses [16], although Ricci et al. suggest that the mortality rate in untreated patients reaches 100% [12].

A recent study carried out in Taiwan shows a frequency of abscesses of the psoas muscle of 2.5 cases per year [19]. In the past, the psoas abscess was mainly caused by tuberculosis of the spine (Pott's disease), but, with the decline of the prevalence of infections caused by mycobacterium tuberculosis, the main pathogens associated with the abscesses of the psoas muscles are those that are related to the diseases of the digestive tract. While the primary abscesses are caused in about 88% of the cases by* Staphylococcus aureus* [12], the secondary abscesses of the iliopsoas are caused by* Streptococcus* species in 4.9% of the cases and* E. coli* in 2.8% [12].

A relatively rare event (Table 3) is instead the abscessual formation of the psoas muscle caused by perforation of a colon cancer (Table 4) with an incidence estimated between 0.3 and 0.4% [20], while the perforation incidence of the cancer colon varies from 3 to 10% [21].

The psoas muscles abscess by perforation of the descending colon cancer may arise or be less with a fistula, in about 15% of the cases [28]. In our case the abscess and the fistula were present. Michowitz et al. proposed a clinical classification of complicated perforations by colon cancer that can be divided into (1) free drilling with loss of intestinal material in peritoneal cavity; (2) drill covered with local training of an abscess; (3) perforation in a neighboring organ or formation of a fistula [29]. Even if the mechanism of formation of the abscess remains unclear, the loss of intestinal contents from perforated cancer may lead to different unusual clinical presentations as retroperitoneal, subcutaneous, and/or perinephric abscesses; perirectal abscesses and fistulas; acute appendicitis and appendicular abscess [30].

An accurate preoperative diagnosis of abscess correlated with a perforated colon cancer is extremely complicated, but the execution of laboratory tests that evidence a marked leukocytosis and especially the use of an abdominal CT is very useful in the diagnosis, in a precise location, and in the determination of the boundaries of the lesion and the dissemination of the surrounding soft tissues. In our case, considering the results of laboratory and instrumental tests and considering the patient clinical condition of acute abdomen we decided for emergency intervention. During the exploratory laparotomy the presence of a malignant perforated cancer and the fistula was evaluated which fired the abscess at the level of the left iliopsoas muscle and ipsilateral obturator muscle. The patient improved greatly his clinical conditions as a result of the intervention that it is concluded with a left hemicolectomy accompanied by a drainage and subsequent repeated washing of the large retroperitoneal abscess and by the removal of the tablet peritoneal seat of the perforation and the fistula. The removal en bloc of colonic tumor of the fistula and the wall of the abscess allowed a better oncological efficiency of the intervention. After surgery a target antibiotic therapy, suggested by culture of abscessual liquid positive for* E. coli* and* Citrobacter braakii*, was set.

In conclusion we have treated a case of perforated adenocarcinoma of the descending colon with an unusual formation of an abscess of the left iliopsoas muscle with involvement of the ipsilateral obturator muscle with relative fistula. The accurate preoperative diagnosis of these conditions clinic is extremely complicated because of the fuzzy clinical presentation, despite the ongoing developments in the context of diagnostic imaging. The importance is to focus on the differential diagnosis and keep in mind that a colon carcinoma can lead to a similar clinical condition. In the collection of the history and during the objective exam, signs of the presence of an abscess are the fever, pain, the presence of a palpable mass, and leukocytosis. The use of the CT can be very useful, even if the definitive diagnosis of colic perforated neoplasia may be evident only during surgery. It is good to intervene as soon as possible in similar conditions with a colon resection affected by neoplasia, with the resection of the fistula (if present), and with the drainage of the abscess to reduce significantly the morbidity and mortality associated with this fearful condition.

## Figures and Tables

**Table 1:** Primary iliopsoas abscess can occur in [17].

Intravenous drug abuse	
Diabetes mellitus	
AIDS	
Renal failure	
Immunosuppression	

**Table 2:** Conditions associated with secondary iliopsoas abscess [18].

Gastrointestinal	Crohn's disease, diverticulitis, and appendicitis
Genitourinary	Urinary tract infection, cancer, and extracorporeal shock wave lithotripsy
Musculoskeletal	Vertebral osteomyelitis, septic arthritis, infectious, and sacroiliitis
Vascular	Infected abdominal aortic aneurysm, femoral vessel, and catheterisation
Miscellaneous	Endocarditis, intrauterine contraceptive device, and suppurative lymphadenitis

**Table 3:** Main clinical features of iliopsoas abscess [18].

Flank and/or back and/or abdominal pain	
Fever	
Limp	
Malaise	
Weight loss	
Lump in the groin	

**Table 4:** Summary of some cases of colon cancer complicated with psoas abscess reported between 1990 and 2013.

Case	Age/sex	Pathogen	Antibiotic	Cancer location	Management	Outcome	Reference
1	85/F	*Streptococcus, Prevotella buccae, and Bacteroides *	IV fosfomycin	Right	LDOA	Recovery	Okita et al. [22]
2	27/F	*E. coli *	IV 2nd and 3rd generation cephalosporin and metronidazole	Left	PD	Recovery	Lee et al. [23]
3	67/M	*E. coli and anaerobic bacteria *	IV carbapenem	Left	PD then LDOA	Recovery	Takakura et al. [24]
4	44/M	*Streptococcus agalactiae and Streptococcus anginosus *	IV ciprofloxacin and metronidazole then IV 3rd generation cephalosporin and metronidazole	Left	Left hemicolectomy and drainage of abscess	Recovery	Yang et al. [25]
5	57/M	*Salmonella group * * B, Bacteroides caccae, * *Bacteroides fragilis, * * and Peptostreptococcus indolicus *	IV ampicillin and sulbactam	Bilateral	Bilateral PD	Recovery	Lo et al. [26]
6	76/F	*E. coli and Proteus penneri *	IV imipenem	Right	PD then laparotomy with right hemicolectomy	Recovery	Tsukuda et al. [27]
Our case	67/M	*E. coli and Citrobacter braakii *	Piperacillin/tazobactam + metronidazole	Left	Laparotomy with left hemicolectomy and drainage of abscess	Recovery	

IV: intravenous; PD: percutaneous drainage; LDOA: laparotomy with drainage of the abscess.
